# Work on Orthopædic Surgery

**Published:** 1869-02

**Authors:** 


WORK ON ORTHOPÆDIC SURGERY.—Messrs. D. Appleton & Co. have in press, and will shortly issue, "A Practical Manual of the Treatment of Club-Foot." By Lewis A. Sayre, M. D., Professor of Orthopædic Surgery in the Bellevue Hospital Medical College, etc.

This little work is intended as a manual for practitioners and students, and gives the results of the author's large experience in the treatment of this deformity. The views of the profession regarding the necessity of tenotomy have undergone such modification within the past ten years, that there is a striking need for a work embodying the latest teachings on this subject. Prof. Sayre's work will meet this acknowledged want.

